# Australian Community Pharmacists' Preparedness to Offer and Discuss Hepatitis C Testing and Treatment With Pharmacy Clients: A Representative Cross‐Sectional Survey

**DOI:** 10.1111/jvh.70001

**Published:** 2025-01-21

**Authors:** Elissa Ong, Ting Xia, Rose Laing, Jacqueline A. Richmond, Peter Higgs, Mark Hayes, Joseph S. Doyle, Suzanne Nielsen, Louisa Picco

**Affiliations:** ^1^ Monash Addiction Research Centre, Eastern Health Clinical School Monash University Melbourne Victoria Australia; ^2^ Disease Elimination Program Burnet Institute Melbourne Victoria Australia; ^3^ Department of Public Health La Trobe University Melbourne Victoria Australia; ^4^ Department of Infectious Diseases Alfred Hospital and Monash University Melbourne Victoria Australia

**Keywords:** Australia, community pharmacists, HCV, hepatitis C, pharmacy

## Abstract

The World Health Organisation (WHO) has set goals to eliminate hepatitis C (HCV) as a global health threat by 2030. To meet this goal, Australia must increase testing and diagnosis, including expanding access to care through community pharmacists. This study aims to explore community pharmacists' preparedness to discuss and offer HCV testing and treatment. Australian community pharmacists from four states completed an online anonymous quantitative survey between August and October 2023. Pharmacists were asked about their experiences of, comfort discussing and willingness to host outreach HCV testing or treatment. Predictors of each outcome were examined using logistic regression. In total, 530 pharmacists participated in the study. One in five pharmacists stocked HCV medications (22%), half (48%) were willing/somewhat willing to host an outreach HCV testing and treatment team, while 36% strongly agreed/agreed they were comfortable discussing HCV testing and treatment. Willingness to host an outreach HCV team was associated with pharmacists working in rural/remote settings (95% CI: 1.04–2.35, *p* = 0.032), providing opioid agonist treatment (95% CI: 1.16–2.49, *p* = 0.006) and comfort discussing overdose prevention (95% CI: 1.31–2.80, *p* = 0.001). Pharmacists with ≥ 15 years' experience (95% CI: 0.44–0.94, *p* = 0.022) were less willing to host outreach HCV testing. Females were significantly less comfortable discussing HCV testing (95% CI: 0.45–0.98, *p* = 0.039) compared to males. This is the first Australian study to explore community pharmacists' preparedness to discuss and offer HCV testing and treatment. In light of research showing that community pharmacy models of care can help meet HCV elimination targets, ongoing engagement with pharmacists is needed to increase their preparedness to provide this care.

## 1 Introduction

In 2019, the World Health Organisation (WHO) estimated 58 million people were living with chronic hepatitis C virus (HCV) worldwide, with approximately 1.5 million new infections occurring each year [1]. In Australia in 2022, an estimated 74,400 people were living with HCV [2] and over 300 deaths are attributed to HCV annually [3]. HCV is a global public health concern, resulting in substantial burden on healthcare systems and the economy [4, 5]. In response, the WHO set targets to eliminate viral hepatitis as a global health threat, by 2030 as part of the Global Health Sector Strategy [6]. Treatment for chronic HCV in the form of direct‐acting antivirals was first introduced in 2014 [7] and can cure more than 95% of cases [8]. Direct‐acting antivirals such as Epclusa (sofosbuvir + velpatasvir), Maviret (glecaprevir/pibrentasvir) and Vosevi (sofosbuvir + velpatasvir + voxilaprevir) were subsidised through the Pharmaceutical Benefits Scheme (PBS) in 2016, as an initial step towards eliminating HCV in Australia [9]. While this resulted in an immediate surge in treatment uptake, this has declined in recent years [10]. This highlights the need for more pathways to increase testing and treatment in Australia [11], including different phases of disease elimination [12], if we are to meet WHO targets.

HCV treatment in Australia was initially specialist‐led, however, there has been a shift to nonspecialist treatment models provided through general practitioners and nurse practitioners [13, 14]. A global systematic review supports decentralisation of HCV care away from specialists in tertiary care settings towards nonspecialists in community settings, including community pharmacies [15]. Additional systematic reviews have specifically demonstrated the feasibility and impact of shifting HCV care into community pharmacy settings [16, 17]. Similarly, pharmacist‐led models of HCV care in the United States and the United Kingdom [18, 19, 20, 21] have demonstrated effectiveness in terms of HCV case detection, treatment uptake and cure [22, 23, 24].

Community pharmacists are a valuable yet often underutilised resource regarding HCV treatment [24]. They have the capacity and expertise to identify drug interactions, improve medication adherence and provide advice regarding side effect management [25]. They can also have established relationships with marginalised populations who are disproportionately affected by HCV and have lower treatment uptake, such as people who inject drugs [26]. For example, through opioid agonist treatment programmes and needle and syringe provision [27], community pharmacists are strongly positioned to have regular contact with people who inject drugs, who may not otherwise be engaged in health services in tertiary or primary care settings [28].

While the international literature supports pharmacist‐led models of HCV care [18, 19, 20, 21, 22, 23, 24], HCV testing is currently not routine practice in Australian community pharmacies. Factors impacting uptake such as familiarity, perceptions, comfort and willingness to perform tasks are important predictors of successful uptake of new pharmacy practices [29, 30], yet it is unclear if and how these may impact community pharmacists' preparedness to offer HCV testing and treatment or if pharmacists are recommending HCV testing to their clients. Furthermore, if pharmacists are to provide expanded access to care, regulatory change may be needed; however, it is currently unknown if pharmacists are prepared to offer such care. To address this gap, this study seeks to: (i) determine the extent to which community pharmacies stock HCV medications and (ii) explore correlates of community pharmacists' willingness and comfort to discuss and offer HCV testing and treatment. A secondary aim is to explore pharmacists' preferences about the content and format of a possible HCV information toolkit to support discussions on HCV within pharmacy settings in the future.

## 2 Methods

### 2.1 Design

Survey data were collected via an online anonymous survey, administered via Qualtrics, an online survey tool, which explored a range of harm reduction services offered within community pharmacy settings.

### 2.2 Setting

This study involved community pharmacists from four Australian states; New South Wales, Queensland, Victoria and Western Australia. These four states were chosen as they are the most populous states, representing 88% of the Australian population [31] and represent the states with the highest rates of notified HCV cases in 2022 [32]. In Australia, there are over 5900 community pharmacies [33]. Community pharmacies are usually private businesses that provide government‐subsidised medicines and clinical services. Pharmacists can be approached for free health advice, without an appointment, often making them the first contact point with the primary healthcare setting. This combined with their accessibility in terms of location and their extended hours makes them the most accessible healthcare provider in Australia [34]. Community pharmacists are crucial to the delivery of primary healthcare, providing education and safely supplying medicines, in addition to health promotion, screening and referral to other healthcare providers [34].

### 2.3 Participants

**FIGURE 1:**
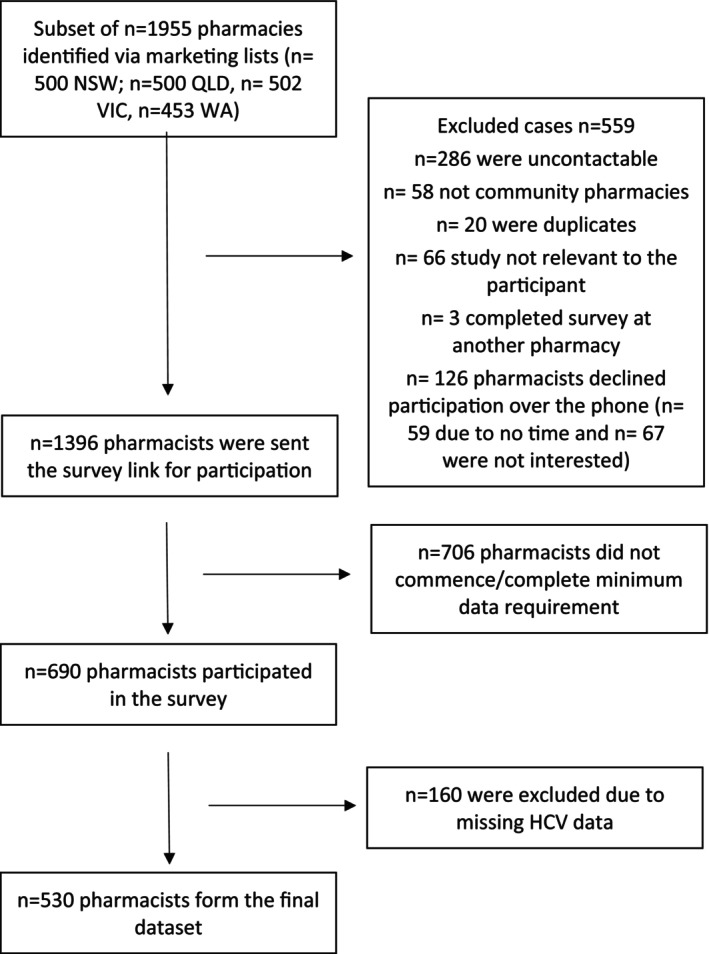
Flow diagram of participation.

To establish a representative sample of community pharmacies in each state, the study used two publicly available pharmacy marketing lists: Australian Marketing and Maven Marketing List. The process of pharmacy selection included combining the two lists and removing duplicates and noncommunity pharmacy listings. Individual state pharmacy lists were randomised using Microsoft Excel formula ‘= rand()’, and a subset of approximately 500 community pharmacies per state were approached (See Figure 1) based on response rates of 43% from an earlier pharmacy study [35] and sample size estimates conducted to ensure there was a sufficient sample size to explore key covariates. A similar number of pharmacies from each state were approached to allow for comparisons across states and to examine correlates in HCV willingness and comfort between jurisdictions.

### 2.4 Procedure

Phone calls were made to a total of 1955 pharmacies, across the four states, between August and October 2023. One pharmacist per pharmacy was invited to participate to remove clustering issues, with the pharmacist in charge selected as they are the pharmacist who is responsible for the overall supervision and operations of the pharmacy. They were chosen as they were considered to have the most accurate picture of the pharmacy setting and greater influence over medication stocking and other practices within the pharmacy. When a pharmacist was uncontactable after three attempts, attempts to contact them ceased. Similarly, where a pharmacist did not express interest in participating, they did not receive additional follow‐up phone calls. Upon expressing interest in the survey, pharmacists were emailed the survey link to their preferred email address. Participants' contact details including their provided email address were stored within an online call log, separate from survey responses to maintain anonymity. Following the initial email with the survey link, reminder emails were sent after 1 and 2 weeks to encourage participation. To incentivise completion of the online surveys, participants could enter an iPad prize draw upon completion of the survey. Identifiable contact information required to enter the prize draw was stored in a separate unlinked database. The study was approved by the Monash University Human Research Ethics Committee (No. 36459).

### 2.5 Survey Questions

Established survey questions that have been used in Australian community pharmacy research were used to guide and inform the current survey [36, 37]. Survey questions were largely drawn from existing surveys that had undergone cognitive testing with pharmacists [35], a process designed to improve the quality of questions by testing comprehension, retrieval, judgement and response processes was also used [38]. A description of specific items is provided below and reported in Appendix A.

#### 2.5.1 Pharmacist and Pharmacy‐Related Characteristics

The survey commenced by asking demographic questions including participant's age, gender and years of experience as a pharmacist. Pharmacy‐specific data were also included such as state, geographic location, operating days and times and whether the pharmacy was independent or part of a pharmacy chain. As the survey was about broader harm reduction services offered in pharmacy practice, questions relating to whether the pharmacy provides services such as a needle and syringe programme, naloxone (which is available free of charge without a prescription from community pharmacies) and opioid agonist treatment (e.g., methadone and buprenorphine, which are covered under the national PBS) were included, and have been used in previous surveys among similar populations [36, 37].

#### 2.5.2 HCV‐Related Questions and Outcome Measurements

A series of HCV‐related questions were incorporated into the survey, whereby pharmacists were asked whether they stocked HCV medications, how frequently they had dispensed these medications in the past 3 months and how often they recommend HCV testing to clients. As providing HCV testing in community pharmacies is not routine practice in Australia and is rapidly evolving, these questions were included to better understand if and how often, these tasks were performed. As no validated measures of pharmacists' comfort and/or willingness were available, questions were developed based on previously established questions utilised within a similar sample [37]. Pharmacists were asked to indicate their willingness to have an outreach HCV testing and treatment team provides onsite testing and treatment, familiarity and comfort with providing treatment and acceptability of developing and using HCV information via an educational ‘toolkit’ for pharmacists to better aid discussions. All responses were measured on a 5‐point Likert scale. Participants were asked about their preference for the content and format of the toolkit (See Appendix A).

### 2.6 Statistical Analysis

Data cleaning and analyses were conducted using IBM SPSS, version 29.0. Descriptive statistics were used to summarise pharmacist and pharmacy characteristics as well as to determine the frequency of endorsement of HCV‐related questions.

To examine factors relating to community pharmacists' willingness and comfort in discussing and offering HCV testing and treatment, 5‐point Likert scale measurements were recoded into binary variables for further logistic regression. ‘Willing’ and ‘Somewhat willing’ were coded as 1 for willingness questions, while ‘undecided’, ‘not really willing’ and ‘not willing’ were coded as 0. A similar approach was adopted for the comfort question, with ‘Strongly agree’ and ‘Agree’ being coded as 1, and ‘neither agree nor disagree’, ‘disagree’ and ‘strongly disagree’ being coded as 0. Pharmacist and pharmacy‐related characteristics variables were entered into the logistical regression models. The measure of effect was reported in odds ratio (OR) for logistic models, with statistical significance being set at *p* < 0.05.

## 3 Results

### 3.1 Sample Characteristics

In total, 530 pharmacists provided data relating to HCV, yielding a response rate of 38%. A similar proportion of male and female pharmacists were represented (male, *n* = 275, 51.9%), with a mean of 15 years of experience working as a pharmacist (SD = 11.3). Just over half of the pharmacists worked in capital cities (*n* = 288, 54.3%), two‐thirds worked at a pharmacy chain or banner group (*n* = 354, 66.8%) and most worked in pharmacies open 7‐days a week (*n* = 326, 61.5%). More than half the participants work in a pharmacy that offered needle and syringe programmes (*n* = 299, 56.4%) and stocked naloxone (*n* = 321, 60.6%) and just under half provided opioid agonist treatment (*n* = 250, 47.2%) (See Table 1).

**TABLE 1:** Sample characteristics.

	*N* (%)
Gender	
Male	275 (51.9%)
Female	252 (47.5)
Age	
21–30	126 (23.8)
31–40	207 (39.1)
41–50	105 (19.8)
51–60	59 (11.1)
> 60	33 (6.2)
Years of experience	
1–5 years	113 (21.3)
6–10 years	111 (20.9)
11–15 years	117 (22.1)
> 15 years	189 (35.7)
State	
New South Wales	118 (22.3)
Queensland	160 (30.2)
Victoria	147 (27.7)
Western Australia	105 (19.8)
Location	
Capital city	288 (54.3)
Urban	91 (17.2)
Rural	112 (21.1)
Remote	39 (7.4)
Type of pharmacy	
Independent pharmacy	176 (33.2)
Chain pharmacy/others	354 (66.8)
Number of days open	
7 days	326 (61.5)
< 7 days	204 (38.5)
Pharmacy hours	
Business hours	410 (77.4)
Extended hours	120 (22.6)
Service provision	
Naloxone	
Yes	321 (60.6)
No	209 (39.4)
Opioid agonist treatment	
Yes	250 (47.2)
No	280 (52.8)
Needle and syringe programme	
Yes	299 (56.4)
No	231 (43.6)

### 3.2 HCV‐Related Information

One in five pharmacists indicated they stocked HCV medications within their pharmacy (*n* = 117, 22%), of which most had supplied HCV medications between one and five times in the past 3 months (*n* = 69, 59%). Most pharmacists (*n* = 308, 58%) indicated they never recommend HCV testing to clients, 17.2% (*n* = 91) rarely recommend HCV testing and only 5.3% (*n* = 28) indicated they do this frequently or occasionally.

Almost half of pharmacists (48%) were willing or somewhat willing to have an outreach HCV testing and treatment team available within their pharmacy, while over a third were undecided (36%). Just over a third (*n* = 190, 36%) agreed or strongly agreed that they were comfortable discussing HCV testing and treatment with their clients, while the majority were undecided (43%) (Table 2).

**TABLE 2:** Hepatitis C‐related descriptive information.

	*N* (%)
Stock HCV medications	
Yes	117 (22.1)
No	413 (77.9)
Frequency of supplying HCV medications in the past 3 months^a^	
≤ 5 times	102 (87.9)
> 6 times	14 (12.1)
Comfort in discussing HCV testing and treatment with pharmacy clients^b^	
Strongly agree	40 (7.6)
Agree	150 (28.4)
Undecided	229 (43.3)
Disagree	80 (15.2)
Strongly disagree	29 (5.5)
Frequency of recommending HCV testing to clients^b^	
Frequently	1 (0.2)
Occasionally	27 (5.1)
Rarely	91 (17.2)
Very rarely	101 (19.1)
Never	308 (58.3)
Willingness to host a free HCV treatment and testing outreach service^b^	
Willing	149 (28.2)
Somewhat willing	103 (19.5)
Undecided	195 (36.9)
Not really willing	47 (8.9)
Not willing	34 (6.4)
HCV toolkit content^c^	
Background information on HCV	437 (82.5)
Options for diagnosis	404 (76.2)
Options for referral pathways	456 (86.0)
Treatment options	439 (82.8)
Others	10 (1.9)
HCV toolkit format^c^	
Written one‐page summaries	434 (81.9)
Online webpage with links	308 (58.1)
Podcasts to listen for pharmacists' information	145 (27.4)
Podcasts to provide to people in the pharmacy	70 (13.2)
Brief animations for pharmacists' education (e.g., 2–5 min YouTube video)	220 (41.5)
Brief animations to provide to people in the pharmacy (e.g., 2–5 min YouTube video)	159 (30.0)
Others	7 (1.3)

*Note:* HCV—Hepatitis C.

^a^ Missing data *n* = 1.

^b^ Missing data *n* = 2.

^c^ Multiple responses can be selected.

Most pharmacists indicated that if an HCV information toolkit were available, they would use the resources relating to background information on HCV (*n* = 437, 82.5%), diagnosis (*n* = 404, 76.2%), referral pathways (*n* = 456, 86%) and treatment options (*n* = 439, 82.8%). The preferred format was a hard copy (e.g., one‐page summary/flyer) (*n* = 434, 81.9%), followed by online (*n* = 308, 58.1%).

### 3.3 Correlates of Pharmacists' Willingness to Host Outreach HCV Testing and Treatment

Pharmacists working in rural or remote settings were more willing to host HCV testing and treatment outreach programmes (OR 1.50, 95% CI: 1.04–2.35, *p* = 0.032) compared to those in capital cities or urban settings.

Pharmacists with 15 years or more of pharmacy experience were less willing to host HCV testing and treatment outreach programmes (95% CI: 0.44–0.94, *p* = 0.02), compared with pharmacists with less than 15 years of experience (Table 3). Compared with pharmacists in New South Wales, pharmacists in Queensland (95% CI: 0.22–0.649, *p* = 0.0001) and Victoria (95% CI: 0.26–0.76, *p* = 0.003) were less willing to host HCV outreach programmes.

**TABLE 3:** Correlates of willingness to host an outreach HCV testing and treatment team.

	Odds ratio	*p*	95% CI
Gender			
Male	1.000		
Female	0.828	0.321	0.571 1.202
Years of experience			
< 15 years	1.000		
≥ 15 years	**0.642**	**0.022**	**0.439 0.938**
State			
NSW	1.000		
QLD	**0.375**	**0.0001**	**0.216 0.649**
VIC	**0.446**	**0.003**	**0.262 0.757**
WA	0.584	0.069	0.327 1.044
Geographic location			
Capital city/urban	1.000		
Rural/remote	**1.562**	**0.032**	**1.039 2.349**
Pharmacy type			
Chain/banner	1.000		
Independent/other	1.429	0.081	0.957 2.134
Comfort discussing overdose prevention with illicit opioid use			
Uncomfortable	1.000		
Comfortable	**1.918**	**0.001**	**1.313 2.799**
Stock naloxone			
No	1.000		
Yes	1.390	0.099	0.940 2.055
Provide OAT			
No	1.000		
Yes	**1.705**	**0.006**	**1.165 2.495**
Provide needle and syringe programme			
No	1.000		
Yes	1.142	0.516	0.765 1.705
Intercept	0.732	0.295	0.408 1.313

Abbreviations: NSW, News South Wales; OAT, opioid agonist treatment; QLD, Queensland; VIC, Victoria; WA: Western Australia. Bold font denotes significance (*p* value < 0.05).

### 3.4 Correlates of Pharmacists' Comfort Discussing HCV Testing and Treatment

Females had reduced odds of being comfortable discussing HCV testing (95% CI: 0.45–0.98, *p* = 0.04) compared to males, while community pharmacists who were comfortable discussing overdose prevention with clients who use illicit drugs had 3.1 times higher odds of being comfortable discussing HCV testing (95% CI: 2.12–4.66, *p* = 0.0001) (Table 4).

**TABLE 4:** Correlates of comfort discussing HCV testing and treatment.

	Odds ratio	*p*	95% CI
Gender			
Male	1.000		
Female	**0.667**	**0.039**	**0.454 0.979**
Years of experience			
< 15 years	1.000		
≥ 15 years	1.189	0.381	0.807 1.754
State			
NSW	1.000		
QLD	0.722	0.253	0.413 1.263
VIC	0.792	0.392	0.464 1.352
WA	0.956	0.881	0.532 1.718
Geographic location			
Capital city/urban	1.000		
Rural/remote	1.220	0.350	0.804 1.850
Pharmacy type			
Chain/banner	1.000		
Independent/other	0.996	0.986	0.660 1.504
Comfort discussing overdose prevention with illicit opioid use			
Uncomfortable	1.000		
Comfortable	**3.143**	**0.0001**	**2.120 4.659**
Stock naloxone			
No	1.000		
Yes	1.052	0.808	0.701 1.578
Provide OAT			
No	1.000		
Yes	1.316	0.174	0.886 1.954
Provide needle and syringe programme			
No	1.000		
Yes	0.954	0.821	0.632 1.438
Intercept	0.331	0.000	0.180 0.611

Abbreviations: NSW, News South Wales; OAT, opioid agonist treatment; QLD, Queensland; VIC, Victoria; WA: Western Australia. Bold font denotes significance (*p* value < 0.05).

## 4 Discussion

This is the first Australian study to explore correlates of community pharmacists' preparedness to offer and discuss HCV testing and treatment, with results revealing moderate willingness and comfort. Examples of pharmacist‐led models of HCV care have been developed elsewhere, with pharmacists conducting various testing modalities including rapid antibody tests, dried blood spot tests and point‐of‐care tests. A Scottish study demonstrated the capabilities of pharmacists to prescribe direct‐acting antivirals [39], while a US study adopted a collaborative treatment model involving physicians and pharmacists [20]. A recent systematic review of hepatitis testing in community pharmacies found clear benefits of providing the entire care cascade from testing through to treatment in pharmacy settings [16]. Considering the high accessibility of community pharmacists in Australia and internationally, the current findings may be of significance in a number of countries. Globally, few countries have developed scalable pharmacist‐led models of HCV treatment, yet many countries have the workforce and infrastructure to provide this model of care. The Australian national HCV strategy highlights the need for increased provision of care in pharmacy settings, and the current findings can help inform implementation and expand access, which in turn may have an international impact, as other countries seek to scale up HCV testing in similar ways.

Only one in five pharmacies currently stock HCV medications. Despite this, half of pharmacists indicated they were willing to host an outreach HCV testing and treatment service (47.7%), while just over a third (36%) were comfortable discussing HCV testing and treatment with their clients. These findings may be explained by known barriers to HCV treatment and testing in community pharmacy settings in other countries such as the United States and Canada, including appropriate remuneration for pharmacists, staffing, time constraints, financial risk associated with upfront costs for ordering in high‐cost medicines and integrating processes into existing workflow and stigma [40, 41]. Contact‐based interventions are designed to improve attitudes and reduce stigma [42, 43] and therefore hosting an outreach HCV service, and having greater contact with people living with HCV, may result in increased comfort in discussing HCV testing and treatment with clients.

Findings suggest moderate willingness and comfort to discuss and provide HCV testing and treatment, suggesting there is considerable scope to provide additional support, education and training, to increase pharmacists' confidence in performing these tasks. Pharmacists were overwhelmingly positive about an HCV information toolkit, which suggests that resources such as this could support pharmacists in discussing HCV testing and treatment. Furthermore, providing adequate training and education for pharmacists is essential to support this model of care and increase preparedness to offer such services [44] and should be a focus among less willing pharmacists. The National Hepatitis C 2023–30 Strategy [45] identifies the need for innovative, multidisciplinary models of care, highlighting pharmacy‐based services as a priority focus. The current findings can help inform where the greatest support is needed to achieve the WHO HCV elimination targets.

Various characteristics were associated with increased willingness to host HCV testing and treatment outreach programmes. Pharmacists in rural and remote settings had higher odds of being willing to host an HCV testing and treatment outreach programme. This is an important finding, given that in Australia, the prevalence of HCV (as a proportion of the population) is highest in rural and remote locations, while treatment and uptake of care are lowest [45], which may partially be explained by less accessible tertiary or specialist treatment in these areas. Given that, even in rural and remote settings, two‐thirds of Australians are within 2.5 km of a pharmacy, combined with increased willingness among these pharmacists, offering outreach HCV treatment within rural and remote pharmacies may be an ideal setting to improve access to HCV treatment and help ensure more equitable access to treatment.

Providing opioid agonist treatment almost doubled the odds of pharmacists being willing to host HCV testing and treatment outreach programmes, as did comfort discussing overdose prevention with clients who use illicit drugs. A 2023 global systematic review on the prevalence and related harms associated with injecting drug use reported the prevalence of HCV among people who inject drugs was 38.8% [46]. Despite high HCV infection rates, this high‐risk population faces a myriad of obstacles when trying to engage with HCV care, resulting in low treatment uptake [47]. Leveraging existing services that are already being accessed by people who use drugs, including opioid agonist treatment via community pharmacies, may be one solution to reach this population. Existing research has demonstrated that pharmacies providing opioid agonist treatment have taken advantage of existing relationships, trust and rapport with clients receiving opioid dependence treatment to increase engagement and improve compliance with HCV treatment, retention and uptake [48, 49]. Offering outreach HCV testing within these pharmacies will help to ensure this undertreated population is a priority of future HCV models of care and is in line with Australia's national HCV policy [45].

More years of pharmacy experience was associated with reduced odds of being willing to host HCV outreach services and has also been associated with negative attitudes towards people who inject drugs [50], perceptions of prescription drug misuse, addiction and diversion [51] and decreased substance use disorder knowledge scores [52]. Workplace culture has also been shown to impact the success of new programmes and the integration of novel processes into healthcare settings [53] and therefore pharmacists with more years of experience may have more entrenched attitudes and ingrained culture. Ongoing and regular education involving people with lived and living experience of injecting drug use and/or HCV may be needed to address this finding [54].

### 4.1 Strengths and Limitations

This is the first Australian study to explore community pharmacists' preparedness to offer HCV testing within community pharmacies. Given the global need to increase access to HCV treatment, findings that support scale‐up of HCV testing and treatment in Australia may have broader relevance to other countries, similarly looking to expand access to care. Robust recruitment methods were adopted to ensure representative samples were recruited in each of the four included states, with pharmacies representing 11% of all Australian community pharmacies and yielding a 38% response rate. However, the following limitations should be considered. The current study only included pharmacists' perspectives and did not assess clients' or other healthcare professionals' perspectives. Desirability bias may have impacted some responses; however, as the survey was anonymous, this will have reduced the impact of this on the results. To our knowledge, no validated scales measuring HCV comfort and willingness were available, therefore we developed these questions based on questions used in earlier pharmacy surveys [37, 55], with input from pharmacists and other HCV clinicians. Furthermore, as comfort and willingness to offer and discuss HCV testing and treatment were captured quantitatively in this study, further qualitative research exploring these concepts and possible barriers in addition to validating measures of HCV comfort and willingness is warranted.

## 5 Conclusion

This study revealed that Australian pharmacists were generally willing to offer outreach HCV testing and treatment, however, they were less comfortable discussing HCV testing and treatment and therefore may require more resources and support. The current national HCV strategy [56] identifies community pharmacy‐based models of care as a key way to achieve HCV elimination targets; however, these findings demonstrate ongoing engagement with pharmacists is needed to support implementation of such care models.

## Conflicts of Interest

JSD discloses funding (to his institution) from Gilead Sciences and Abbvie for research, and honoraria (to his institution) from Abbvie.

## Data Availability

The data that support the findings of this study are available on request from the corresponding author. The data are not publicly available due to privacy or ethical restrictions.

## Appendix A

### A.1

**Q1. What is your current age?**

< 21 years

21‐30 years

31‐40 years

41‐50 years

51‐60 years

>60 years

**Q2. What is your gender?**

Male

Female

Non‐binary/gender diverse

Other, please specify____________________

**Q3. How many years have you been a registered practicing pharmacist (including intern year or provisional registration)? __ years (scroll down of all year options)**

**Q4. In what state is the pharmacy you work at located? (If you work in more than one state, please refer to the state in which you were contacted about this survey)**

New South Wales

Queensland

Victoria

Western Australia

**Q5. What is your pharmacy's geographic location?**

Capital city (Melbourne, Sydney, Brisbane, Perth)

Other urban centre (population >100,000 e.g Geelong, Ballarat, Wollongong, Newcastle, Cairns, Townsville)

Rural location (population between 5,001 and 99,999)

Remote (population < 5000)

**Q6. What type of pharmacy do you work in?**

Single independent pharmacy

Small chain or banner group (2‐9 branches)

Large chain or banner group e.g Amcal, Chemist Warehouse, Priceline (>10 branches)

Other, please specify ________

**Q7. How many days a week is your pharmacy open?**

1 2 3 4 5 6 7

**Q8. What are the average opening hours of the pharmacy you work in?**

Business hours (e.g. somewhere between 8:00am‐6:00pm)

Extended hours (beyond business hours) up to 24 hours a day

**PHARMACY AND SERVICES RELATED QUESTIONS**

**D1. In the past week, how often did you receive prescriptions for opioids at your pharmacy?**

Once per week

Multiple times a week

Around once per day

Several times a day

More than ten times a day

**D2. In general, how comfortable do you feel intervening when you are concerned about an opioid prescription?**

Very uncomfortable

Uncomfortable

Comfortable

Very comfortable

**D3. How comfortable are you discussing overdose prevention and naloxone with patients who are prescribed opioids?**

Very uncomfortable

Uncomfortable

Comfortable

Very comfortable

**D4. How comfortable are you discussing overdose prevention and naloxone with patients who use illicit drugs (e.g. heroin)?**

Very uncomfortable

Uncomfortable

Comfortable

Very comfortable

**D5. Do you stock naloxone in your pharmacy?**

Yes

No

**D6. Do you offer Medication Assisted Treatment for Opioid Dependence (MATOD) at your pharmacy? (i.e methadone and buprenorphine)**

Yes (Go to Q27a)

No (Go to Q28)

**D7. If yes, which of the following do you provide (tick all that apply):**

Methadone dosing on site

Buprenorphine (sublingual) dosing on site

Long acting injectable buprenorphine supply to external services

Long acting injectable buprenorphine administration on site

Other form of MATOD __________________(please provide details)

**D8. Do you offer a needle and syringe program at your pharmacy?**

Yes

No

**D9. Do you stock Hepatitis C treatment/medications in your pharmacy?**

Yes (Go to Q29a)

No (Go to Q30)

**D10 If yes, how many patients have you supplied Hepatitis C medications to in the past 3 months? _____**

**D11. To what extent do you agree with the following: I am comfortable discussing Hepatitis C testing and treatment with people in the pharmacy where I work**

Strongly Agree

Agree

Undecided

Disagree

Strongly Disagree

**D12. If an information ‘toolkit’ was developed to support discussions on Hepatitis C in the pharmacy, which of the following would you use (tick all that apply):**

Background information on Hepatitis C

Information on options for diagnosis

Information on the options for referral pathways

Information on the options for treatment

Other, please specify __________________________

**D13. Which formats would these resources be useful in (tick all that apply):**

Written one page summaries

Online webpage with links

Podcasts to listen to for your own education

Podcasts to provide to people in the pharmacy

Brief animations to view for your own education (e.g. 2‐5 min youtube video)

Brief animations to provide to people in the pharmacy (e.g. 2‐5 min youtube video)

Other, please specify ______________________________________

**D14. How often do you currently recommend Hepatitis C testing to clients?**

Very Frequently

Frequently

Occasionally

Rarely

Very Rarely

Never

**D15. If an outreach Hepatitis C testing and treatment team (based at a local or state service) offered to provide free testing and treatment in your pharmacy, how willing would you be to accept that service?**

Willing

Somewhat willing

Undecided

Not really willing

Not willing

**D16. If you are interested in hosting a free Hepatitis C testing and treatment service, how often could you host them?**

• Weekly

• Monthly

• Once every few months

• Once every 6 months

• Once a year or less
